# Does Prison Crowding Predict Higher Rates of Substance Use Related Parole Violations? A Recurrent Events Multi-Level Survival Analysis

**DOI:** 10.1371/journal.pone.0141328

**Published:** 2015-10-22

**Authors:** Michael A. Ruderman, Deirdra F. Wilson, Savanna Reid

**Affiliations:** 1 College of Osteopathic Medicine, Touro University California, Vallejo, California, United States of America; 2 Public Health Program, Touro University California, Vallejo, California, United States of America; 3 Department of Epidemiology, University of Nevada, Las Vegas, Nevada, United States of America; Catholic University of Sacred Heart of Rome, ITALY

## Abstract

**Objective:**

This administrative data-linkage cohort study examines the association between prison crowding and the rate of post-release parole violations in a random sample of prisoners released with parole conditions in California, for an observation period of two years (January 2003 through December 2004).

**Background:**

Crowding overextends prison resources needed to adequately protect inmates and provide drug rehabilitation services. Violence and lack of access to treatment are known risk factors for drug use and substance use disorders. These and other psychosocial effects of crowding may lead to higher rates of recidivism in California parolees.

**Methods:**

Rates of parole violation for parolees exposed to high and medium levels of prison crowding were compared to parolees with low prison crowding exposure. Hazard ratios (HRs) with 95% confidence intervals (CIs) were estimated using a Cox model for recurrent events. Our dataset included 13070 parolees in California, combining individual level parolee data with aggregate level crowding data for multilevel analysis.

**Results:**

Comparing parolees exposed to high crowding with those exposed to low crowding, the effect sizes from greatest to least were absconding violations (HR 3.56 95% CI: 3.05–4.17), drug violations (HR 2.44 95% CI: 2.00–2.98), non-violent violations (HR 2.14 95% CI: 1.73–2.64), violent and serious violations (HR 1.88 95% CI: 1.45–2.43), and technical violations (HR 1.86 95% CI: 1.37–2.53).

**Conclusions:**

Prison crowding predicted higher rates of parole violations after release from prison. The effect was magnitude-dependent and particularly strong for drug charges. Further research into whether adverse prison experiences, such as crowding, are associated with recidivism and drug use in particular may be warranted.

## Introduction

High incarceration rates in the United States have raised serious public health concerns, both related to prison experience as an exposure to stress, injury and infectious disease, and related to imprisonment as a negative outcome of adverse social determinants of health in the environment [1–5]. Whereas advocates of high incarceration rates cite rational choice theory to predict that consistent punishment will reduce crime rates, some observers of disadvantaged communities see the criminal justice measures taken to deter crime as exacerbating poverty traps that are conducive to high crime rates [6]. Crowding overextends prison supervision, allowing violence against inmates to go unchecked. It also dilutes access to drug treatment resources. Trauma and deficient mental health services are both risk factors for substance use disorders and other psychiatric illnesses. Prisons also promote the acquisition and spread of infectious diseases through crowding, and this is especially true for drug-related HIV and hepatitis C infections and crowding-related tuberculosis infections [6, 7]. In California, the contribution of severe crowding to violence and grossly substandard inmate healthcare warranted legal intervention by the federal government; the U.S. Supreme Court mandated that the state reduce its incarceration rates to redress crowding problems [8].

This study examines prison crowding as a risk factor for recidivism. Crowding is defined as occupancy rate, which can be found by dividing the designed maximum capacity into the number of prisoners housed in an institution, and multiplying this by 100. Records from the California Department of Corrections and Rehabilitation (CDCR), from 1991 through 2010, show the mean rate occupancy across all state institutions was 186.96% [9]. Some prisoners have spent the entirety of their sentences in converted reception centers operating at an occupancy rate of over 300% [10]. Furthermore, in the last 30 years California's general prison population increased 572%, and its parole population grew by 708% [11].

Crowding-related stress is associated with both aggression and impaired executive function in both children and adults [12, 13, 14]. Short-term crowding has been shown to lead to more competitive behavior in children and adolescents, particularly for males, in a study examining cooperative and competitive play after exposure to crowding [15]. In adults, residential crowding stress has been linked to negative affect, social tension, learned helplessness and behaviors that are more competitive, reactive, and involved with reestablishing control [16, 17, 18]. The enforced helplessness characteristic of prison life has been shown to exacerbate crowding-related behavior problems on several dimensions [16, 17, 18]. This study's hypothesis, that prison crowding predicts higher rates of recidivism, assumes that after an exposure to crowding-related stress, an adult's behavior and cognitive coping skills continue to be negatively affected by that experience, an assumption that has not yet been directly tested but which finds indirect support in some previous research [13, 15].

This study's outcome variable, recidivism, is associated with chronic socioeconomic and psychosocial disadvantage in communities affected by high rates of incarceration. California has some of the highest recidivism rates in the United States, and its unique parole system is a factor [19]. Almost all prisoners are released on parole, usually for three years, with varying levels of monitoring. Recidivism may take any of three forms: (i) parole violations, (ii) new arrests, or (iii) parole revocation (termination of parole). Both parole violations and new arrests are multifactorial and are related to behavioral, socioeconomic, community, and policy-driven conditions [20]. However, parole revocations are decided by an administrative board and are the ultimate determinant of whether a parolee returns to prison [19]. Despite this review, inmates released from California prisons from 2006 to 2007 had an average recidivism rate of over 65%. In addition, among offenders who had been released two or more times, the rate was over 75% [21]. Overall, more than 40% of prisoner admissions in 2010 were from parole violators returning to custody [22].

This study's hypothesis, that prison crowding is associated with higher rates of recidivism, contradicts the conventional wisdom in the field of criminology. Criminology theories, such as rational choice theory, argue that individuals make law violation decisions based on their individual needs relative to the perceived risk of legal consequences [23]. A crude way of applying this theory would hold that adverse prison conditions (such as crowding) should increase perceived risk, therefore decreasing the likelihood of individual recidivism. However, a more fine-grained application of rational choice theory would hold that adverse prison conditions are less salient to parolees facing an opportunity to reoffend than other considerations, such as the perceived risk of arrest and successful prosecution, and the length of sentences associated with the violation in question.

In fact, several studies have actually found that harsher prison conditions were positively associated with recidivism. One of these studies, a study of federal inmates, found that higher levels of security housing (commensurate with increased restrictions) did not reduce recidivism [24]. In addition, a survival analysis of California prisoners randomized into different security level classifications found that the highest level had more than a 30% increased risk of returning to prison compared with the lowest level [25]. Higher security levels often correspond to longer sentences, an important confounder in this analysis. Such sentences lead to less successful reintegration into society after release, and thus contribute to recidivism. However, these studies still raise doubts as to whether harsh prison conditions are crime deterrents, and they support the possibility that crowding could be one factor behind California's high recidivism rates.

If crowding does increase a prisoner's risk of recidivism, this could be explained by the psychosocial stress associated with adverse prison conditions, which may exacerbate decision-making problems (e.g., impulsivity) and problem behaviors (e.g., drug use, aggression) in prison populations. Such a relationship would, necessarily, be mediated by factors overrepresented in prison populations that include mental illness, cognitive dysfunction and intellectual disability, in addition to factors related to former prisoners' social environment. The high prevalence of substance use disorder (SUD) in prison populations may also be a factor in the high rates of drug-related recidivism seen among California parolees [22].

Relatively little research has been done to date on the relationship between prison crowding and recidivism. Insufficient statistical methods and the availability of only aggregate level data hindered early attempts to study the effect of crowding specifically on recidivism [26–27]. One recent individual-level data study of twenty-five thousand Italian former prisoners found that prison crowding was directly associated with recidivism [28]. However, these researchers were unable to measure the effect size with great precision (the upper limit of the confidence interval for their estimated effect size was 0.064, whereas the lower limit was negative and close to 0). Additionally, they did not assess what types of violations were more susceptible to an effect on recidivism.

In one other study of California parolees, investigators found that the more overcrowded a correctional institution was, the less likely parole boards were to return a violator back to prison [29]. Their analysis, however, did not consider crowding as a risk factor for parolees to actually violate parole. It is also unknown how these parole board decisions may have ultimately altered recidivism rates for specific parole violation types.

### Study purpose and rationale

Crowding overextends prison resources, to the detriment of prisoner safety and public health [6]. Running prisons over capacity makes adequate inmate behavior monitoring nearly impossible, leading to increased violence and rape [30–38]. Additionally, overcrowded prisons cannot offer adequate drug treatment or any other medical care [39–41]. Limited access to drug treatment may contribute to higher rates of drug use-related recidivism among parolees with SUD. For example, CDCR inmates who did not take part in drug treatment during and after prison had double the recidivism rates of those who did [22]. This study investigates the potential for a vicious cycle between high incarceration rates and high crime rates, mediated by prison crowding levels.

### Study question

The aim of this study was to evaluate whether parolees exposed to higher levels of prison crowding returned to prison at higher rates than others. Consequently, the research question was as follows: Does prison crowding exposure predict higher rates of parole violations? The hypothesis of this study is that crowding is associated with higher rates of parole violations.

Psychosocial stress is the hypothesized mediator of the effect of crowding on rates of parole violations. However, this study’s statistical inference is limited to the effect of study exposure (prison crowding) on the study outcome (rates of parole violations), with no data on subjects' stress hormone levels (e.g., cortisol) or other clinical measures of stress associated with crowding-related behavior problems. We can look at effect size differences for different types of parole violations (e.g., drug-related charges), but because of the high frequency of missing data on use of drug treatment programs within the study population (26%), and because data on SUD diagnosis were only available at the aggregate level, we are unable to control for history of SUD.

## Methods

### Study population

The study population comprised a random sample of 5% of California’s parolees under the supervision of CDCR between January 1, 2003, and December 31, 2004. Event data were included on 13070 parolees out of the total population of 254468. Rates of parole violations over this two year observation period were calculated using recurrent events survival analysis.

### Data

#### Sources

Data were combined from two sources. The first, maintained by the National Archive of Criminal Justice Data (NACJD), contains the individual level data for all variables except prison crowding level [42]. Survival observations were made weekly to record whether or not a subject violated parole and which type of violation, if any, occurred. Violation events were counted whenever a parolee was observed to have violated a technical or Level I to Level III violation, or absconded. Technical violations include missed appointments with officers, visiting outside the parole region, and other non-criminal offenses. California is one of a few states that returns parolees to prison as a sanction for technical violations. Absconding violations include both cases in which the parolee has left the state's jurisdiction without permission and those in which he or she has simply failed to report for parole supervision. Level I violations are predominantly personal drug offenses, such as drug use, drug possession, failure to register as a drug offender, being under the influence, being drunk in public, etc. Level II violations include drug sales, property offenses, and other non-injury crimes. Level III violations consist of serious and violent crimes. Non-violation events were counted whenever a parolee: (i.) had no violation during an observation, (ii.) was censored, or (iii.) had their parole period end without violation.

The second source of data comprises monthly adult prison population reports hosted on CDCR's official website. Population totals were aggregated at the institutional (prison) level. This study utilized reports for the months of July, 1990, through December, 2002.

#### Aggregating crowding levels

Monthly CDCR population census reports were digitized, compiled, and cleaned. Prisons were then grouped according to geographical region, corresponding with CDCR’s four parole regions based on map plotting. Region I includes the northern part of the state, not including the coast. Region II includes only the coastal regions of Northern California. Region III includes only Los Angeles County. Region IV contains both the coastal and inland areas of Southern California. Weighted averages of prison crowding levels were then calculated for each group of prisons in a parole region over the 150 study months, resulting in 600 weighted measurements of crowding. These continuous values were subsequently grouped into three discrete levels: “low,” “medium,” and “high.” Cut-off levels were based on maximum capacity recommendations from the United States Government Accountability Office and expert testimony [39, 43]. “Low” was defined as less than 190% capacity, “medium” was defined as between 190% and 205% capacity, and “high” was defined as 205% or greater. It was impossible to create a group without any crowding since all Californian prisons were over capacity.

#### Assigning aggregate crowding levels to individual subjects

The crowding exposure level for each subject was determined by the parole region of the parolees’ prison and the crowding level in that region during the month prior to the date of their release from prison. Occasionally, some prisoners (particularly those with medical conditions) are transferred between institutions. However, even for these prisoners, the last thirty days represents a significant portion of total time served (77% of parolees served <5 months during the study) [10]. Release date was calculated by subtracting a given parolee’s days of freedom from the study start date (rounded to the nearest month).

### Data Analysis

#### Software

All data assembly and analysis were performed using *R*, an open source, object-oriented programming language and environment [44]. All time-related data were first made date attributable using the package *Lubridate*, making time calculations feasible [45]. A hot-decking method of data imputation utilizing random recursive partitioning (RRP) for matching was implemented using the *RRP* library [46]. This is a Monte Carlo procedure which generates a proximity matrix for nonparametric missing data imputation, classification, prediction and matching problems [47]. Cox proportional hazard regression models, including stratified and recurrent models, were run using functions from the *Survival* package [48].

Some analysis and adjustments to the data were made by the NACJD prior to this research project, and we cannot describe their work in this paper. The NACJD investigators did have access to the relevant death file, but could not describe how censoring accounted for mortality at the time of our research project. We do know that parolees were censored when their parole period ended without a violation or when the study period ended before they had either violated or completed parole. We also know that there was left-truncation of the data set (for example, almost half the observations used in the final model are for parolees who had been on parole for more than a year before the observation period begins).

#### Rationale for using recurrent events survival models

The standard Cox proportional hazards model is a nonparametric type of survival analysis that allows for adjusted calculations of hazard ratios without needing to express a baseline hazard. However, upon release parolees could have accumulated several parole violations before being returned to prison. Therefore, it was necessary to use a survival analysis model that could accommodate recurrent events within a subject. There are several approaches to recurrent events survival analysis that have been shown to be superior to the standard Cox proportional hazards model [49]. This may be accomplished by using a counting process approach, which considers separate event observations of the same subject as though they were from different subjects using start and stop times for interval truncation [50]. Thus our data for time until parole violation was converted into counting process format (stop/start times) for use in Cox proportional hazards models modified to account for recurrent events with discontinuous event times (time in prison was excluded from time at risk). It should be noted that because this cohort study used recurrent events survival analysis, we measured rates of parole violations rather than time until parole violations, unlike a conventional Cox proportional hazards model.

#### Adjusting variance for within-subject correlation

Robust estimation was used to adjust the variance of all model coefficients to account for within-subject correlation, using a sandwich method similar to that introduced by Lin and Wei [51]. Frailty methods are considered more effective than counting process models with robust error variance estimation at capturing heterogeneity when some subjects are believed to be more prone to accumulating events than others, notably reducing the risk of underestimating effect sizes, but where there is no strong biological relationship between first events and subsequent events, the methods are comparable and in some cases, their results are very similar [50, 52]. Because this technique involves adjusting only the estimated variances and not the regression coefficients for the misspecification of the correlation structure assumed (i.e., the non-independence of recurrent events), the hypothesis test using this method involves evaluating the confidence intervals for effect sizes, rather than the effect sizes themselves [51].

#### Evaluating the proportional hazards assumption

All variables were tested to determine whether the hazard ratios for any two variables in a model were constant. These assumptions were tested using a graphical approach. Specifically, log-log Kaplan-Meier curves were plotted. They satisfied the assumption if the curves were subjectively parallel.

#### Stratified Cox procedure

The models produced in this study included variables that both did and did not satisfy the proportional hazards assumption. That is, the hazard ratios for some variables were not constant over time. A Cox stratified procedure was used to control for both types of variables. This involves stratifying the data by a categorical variable that violates the proportional hazards assumption [53]. One disadvantage of this method is that it does not show how the hazard ratios vary over time.

#### Missing data

Missing data on parole region (hence, exposure to prison crowding), time under observation (days since release), and several of the covariates were imputed only for violation events and censored observations. (Data on non-violation observation weeks are not used in the Cox proportional hazards model). Missing data were less extensive for these events than for the dataset as a whole; the total percentage of imputed values was 11.5% and the highest number of imputed values for a given event was three. The reasons for missing data are unknown and may, therefore, be non-random. A random recursive partitioning (RRP) method for non-parametric matching was used; this is a Monte Carlo procedure which generates a proximity matrix using regression trees for missing data imputation. The algorithm used 250 replications of the procedure, with continuous variables divided into 14 intervals [47]. Missing data were then imputed using a hot-decking method, that is, one that matches persons with missing data to persons with similar data on all other covariates and gives the corresponding value for the missing data [47].

### Baseline characteristics

This study’s target population was predominantly male. Parolee demographics were 30.7% white (non-Latino) and 26.1% Black, despite the fact that there are six times as many non-Latino white people living in the state compared to non-Latino Blacks [54]. The categorical data on race was transformed into a dichotomous variable for the analysis (either Black or non-Black). Approximately 80% of the parolees were between 18 and 44 years of age. About 66% reported having a substance use disorder and over half reported having a comorbid mental health condition [55]. Only aggregate level data were available on substance use disorder, however the final adjusted model does include mental health conditions as a controlled confounder.

The type of offense for which subjects were on parole (not to be confused with the type of parole violation) was handled as a categorical variable, with drug offense as the reference value, as this was the largest category. For race and parole officer race, the reference category was non-Black, and for sex and parole officer sex, the reference category was female. Three striker status refers to a "three strikes law" requiring harsher sentences for those with a second or third serious offense, and in this data set parolees are designated "strikers" if this was their second or third serious offense. Serious offenses are distinguished from crimes such as personal drug use or tax evasion that are unlawful, but do not endanger others. Mental illness status was ascertained from official documentation on parolees, which may in some cases have been incomplete. Parole supervision levels were defined as follows: (1) Minimum service level, communicating with the parole agent primarily through the mail (no drug testing), (2) Controlled service level, a moderate level of supervision that may include services for drug use, mental health problems, education or employment deficits, (3) High control level, where the emphasis in supervision is on detecting or preventing serious criminal activity, (4) High services level, in which high levels of supervision are focused on the service needs of the parolee, and (5) High risk spec/non-spec, in which the parolee is supervised by a parole agent whose caseload is composed entirely of high risk parolees who require a high level of supervision.

The baseline event characteristics are described in Table 1, broken down by crowding exposure level. Chi square statistics were calculated for differences across crowding levels, and Table 1 presents the p-values and degrees of freedom for these statistics. Because these are data on parole violation or non-violation events (not persons), they represent a summation of the most recent data for parolee characteristics with respect to each event for each person (for those variables that may change between events).

**Table 1:** Baseline characteristics of California parolees by prison crowding level.

Variable	Low Crowding	Mid Crowding	High Crowding	Combined	Chi square p-value	Degrees of freedom
	(N = 8081)	(N = 10700)	(N = 3081)	(N = 21862)		
**Demographics**						
Male	89% (7219)	90% (9660)	90% (2772)	90% (19651)	.10	2
Black	32% (2563)	26% (2818)	27% (818)	28% (6199)	.001	2
Mentally Ill	23% (1888)	26% (2762)	26% (816)	25% (5466)	.001	2
Age 18–30	34% (2773)	35% (3766)	38% (1165)	35% (7704)	.003	2
Age >45	15% (1214)	14% (1531)	15% (453)	15% (3198)	.39	2
Three Striker	13% (1060)	15% (1621)	16% (481)	14% (3162)	.001	2
**Offense Type**						
Drug	36% (2869)	36% (3862)	34% (1062)	35% (7793)	.001	2
Violent	20% (1631)	19% (2019)	17% (524)	19% (4174)	.001	2
Property	29% (2316)	31% (3343)	35% (1090)	31% (6749)	.001	2
Other	11% (911)	10% (1062)	10% (323)	11% (2296)	.01	2
Sexual	4% (354)	4% (414)	3% (82)	4% (850)	.001	2
**Number of Prior Imprisonments**						
1^st^ time in prison	36% (2910)	31% (956)	31% (3307)	33% (7173)	.001	2
2^nd^-3^rd^ time	31% (2505)	29% (901)	31% (3296)	31% (6702)	.18	2
≥ 4^th^ time	33% (2666)	40% (1224)	38% (4097)	37% (7987)	.001	2
**Parole Region**						
Region 1	21% (1713)	30% (3191)	0% (0)	22% (4904)	.001	6
Region 2	50% (4045)	4% (419)	0% (4)	20% (4468)	.001	6
Region 3	29% (2318)	28% (3030)	34% (1061)	29% (6409)	.001	6
Region 4	0% (5)	38% (4060)	65% (2016)	28% (6081)	.001	6
**Supervision**						
Level 1	60% (1835)	49% (3984)	53% (5710)	53% (11529)	0.001	2
Level 2	13% (400)	11% (919)	12% (1307)	12% (2626)	0.043	2
Level 3	1% (41)	1% (61)	1% (86)	1% (188)	0.009	2
Level 4	15% (469)	27% (2153)	22% (2315)	23% (4937)	0.001	2
Level 5	11% (336)	12% (964)	12% (1282)	12% (2582)	0.24	2
**Violation Type**						
No Violation	59% (4741)	59% (6262)	67% (2067)	60% (13070)	.001	2
Technical	4% (357)	4% (463)	3% (88)	4% (908)	.001	2
Absconded	11% (907)	13% (1367)	13% (399)	12% (2673)	.002	2
Type I (e.g., personal drug offenses)	10% (792)	10% (1052)	7% (218)	9% (2062)	.001	2
Type II (e.g., drug sales, property crimes)	9% (747)	8% (902)	6% (183)	8% (1832)	.001	2
Type III (serious and violent crimes)	7% (537)	6% (654)	4% (126)	6% (1317)	.001	2
Combined	41% (3340)	41% (4438)	33% (1014)	40% (8792)	.001	2
**Age First Incarcerated**						
<24	28% (2301)	33% (3498)	35% (1064)	31% (6863)	.001	8
24–28	16% (1315)	15% (1615)	16% (486)	16% (3416)	.001	8
29–35	24% (1960)	24% (2582)	22% (687)	24% (5229)	.001	8
36–42	21% (1665)	19% (1999)	18% (561)	19% (4225)	.001	8
>43	10% (840)	9% (1006)	9% (283)	10% (2129)	.001	8
**Parole Officer**						
Male	70% (2145)	77% (6238)	72% (7715)	74% (16098)	.001	2
Black	31% (957)	32% (2552)	27% (2905)	29% (6414)	.001	2

Baseline characteristics of 21 862 events (parole violation or not) for 13 070 parolees according to magnitude of prison crowding exposure prior to release.

Significant differences in exposure to prison crowding were found in most variable groups. These groups included general demographic differences, offense type, parole region, supervision level, and age at first incarceration. Black parolees were more likely than non-Black parolees to have been in a prison with low crowding levels. Mentally ill parolees were more likely to have been in a prison with high crowding levels. Parolees imprisoned for violent crimes were less likely to have been in a prison with high crowding levels, while the opposite was true for those imprisoned for property crimes. Parolees at supervision level 1 were more likely to have been in a prison with low crowding levels, while those at level 4 were more likely to have been in a prison with medium or high crowding levels. Those who had been imprisoned four or more times before were more likely to experience higher levels of prison crowding than those imprisoned for the first time. Only the variable parole region differed dramatically between crowding levels. Specifically, there were no prisons in either parole regions one or two that experienced high levels of crowding. In region four, no prisons experienced low levels of crowding.

#### Adjustment for confounding

Hazard ratios were adjusted for confounding variables and for variables that increased precision. Socioeconomic factors were also considered in our analysis, but were not significant confounders; these variables also had a disproportionately higher frequency of missing values (>40%). No interaction effects were found between the variables. Left-truncation of the dataset was accounted for by controlling for age at first incarceration. A Cox stratified procedure (described above) was used to control for those variables that violated the proportional hazards assumption and those that did not. Controlled variables that satisfied the proportional hazards assumption were sex, race, mental health status, prior offense type, level of supervision, three-striker status, parole officer sex, and parole officer race. Controlled variables that did not satisfy the proportional hazards assumption were sex offender status, parole region, age at first incarceration, age at release, number of prior imprisonments, and number of prior serious offenses leading to imprisonment.

### Ethics Statement

All individual level data received anonymous identifiers and were encrypted for storage when not in use. The National Archive of Criminal Justice Data reviewed and approved the study protocol and data protection plan. Additionally, the study was reviewed and exempted by the Touro University-California Institutional Review Board (IRB) because only non-identifiable data on human subjects were used.

## Results

This study period included 21 862 events that were either classified as violation or non-violation events. The vast majority of the sample, approximately 98%, was observed for a period of 104 to 106 weeks. During the observation period, 8792 parole violations were observed, including technical, criminal, and absconding violations. There were no missing violation values. In unadjusted analysis, parolees had fewer parole violations if they had been imprisoned at high crowding levels for combined parole violation types, but they were more likely to have absconded if they were imprisoned at medium crowding levels.

### Parole Violations

After adjustment for confounding, prison crowding was associated with higher rates of parole violations in a magnitude-dependent manner. Specific types of violations had different effect sizes. From largest to smallest, they were absconding, Type I, Type II, Type III, and technical violations.

### Crude and partially adjusted results

**Fig 1:**
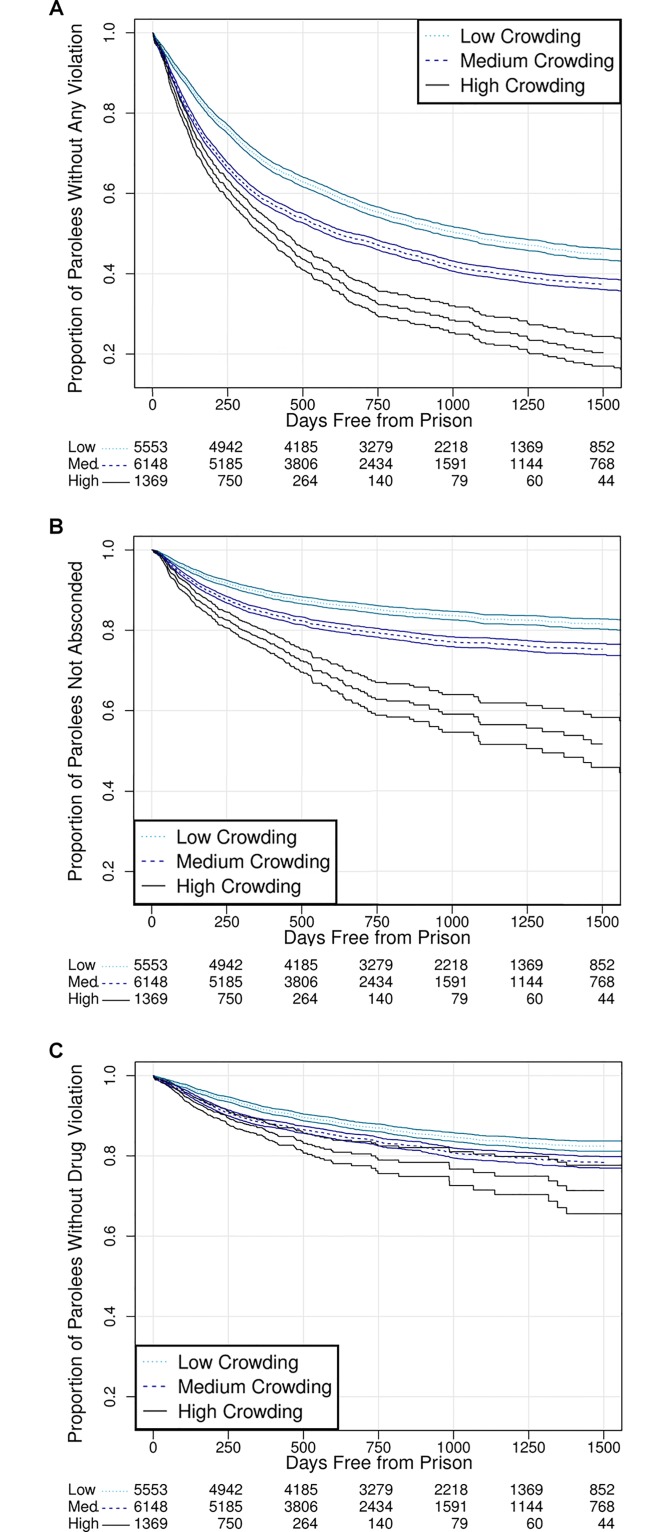
Survival Curves: Days Free from Prison by Crowding Level. Kaplan-Meier survival curves of days free from prison for (A) combined parole violations, (B) absconding violations, and (C) drug use violations. [Note: Curves are bounded by 95% confidence intervals. Adjustments were made only for those variables satisfying the Cox proportional hazards assumption. All curves were robust, with little variation between stratified models.]

Overall, without any adjustments, the proportion of violations among low, medium, and high crowding levels was relatively similar (41%, 41%, 33%, respectively). Partially adjusted Kaplan-Meier curves for all violation types diverged over the 50-month period, most immediately after release, and the effects were positively proportional to crowding level. The largest effects were seen for combined, absconding, and Type I violations (Fig 1).

Fig 1A shows that by the end of the study period, fewer than 20% of parolees from high crowding prisons remained without parole violations, whereas close to 40% of those from moderately crowded prisons, and even more from prisons with low levels of crowding, remained without parole violations. For both combined violations and absconding violations, that is, Fig 1A and 1B, the differences between crowding levels are dramatic within the first 500 days of parole. For level one violations, shown in Fig 1C, close to 80% of parolees remained without a violation at all crowding levels, but the curves do still diverge significantly, with higher rates of parole violations for parolees who had been imprisoned at higher crowding levels.

### Adjusted results for any violation type

After all adjustments, rates of parole violations were 2.28–2.77 times greater for parolees from highly crowded prisons compared to those from prisons with low levels of crowding (HR 2.52, 95% CI: 2.28–2.77). The effect size was 1.52–1.72 times greater when comparing medium crowding prisons with low crowding prisons (HR 1.62, 95% CI: 1.52–1.72). The full model is presented in Table 2. Hazard ratios for controlled confounders are also reported because these provide, in a very limited fashion, indirect information about the relative importance of some uncontrolled confounders (e.g., surveillance bias, socioeconomic status, history of SUD).

**Table 2:** Adjusted* Hazard Ratios for Crowding and Other Predictors of Earlier Parole Violations.

	Parole Violation Type											
	Combined		Absconding		Type I		Type II		Type III		Technical	
	HR	95% CI	HR	95% CI	HR	95% CI	HR	95% CI	HR	95% CI	HR	95% CI
**Prison Crowding**												
High	2.52	(2.28–2.77)	3.56	(3.05–4.17)	2.44	(2.00–2.98)	2.14	(1.73–2.64)	1.88	(1.45–2.43)	1.86	(1.37–2.53)
Medium	1.62	(1.52–1.72)	1.86	(1.66–2.07)	1.69	(1.49–1.91)	1.49	(1.31–1.70)	1.41	(1.21–1.65)	1.45	(1.21–1.74)
Low	1.00	(reference)	1.00	(reference)	1.00	(reference)	1.00	(reference)	1.00	(reference)	1.00	(reference)
**Demographics**												
Male	1.21	(1.12–1.32)	1.07	(0.95–1.20)	1.33	(1.15–1.54)	1.30	(1.11–1.53)	1.30	(1.06–1.58)	1.17	(0.93–1.48)
Black	1.17	(1.11–1.24)	1.13	(1.05–1.23)	1.11	(1.01–1.23)	1.17	(1.05–1.29)	1.21	(1.07–1.36)	1.38	(1.19–1.61)
Mentally Ill	1.46	(1.38–1.55)	1.47	(1.35–1.60)	1.48	(1.34–1.63)	1.59	(1.43–1.76)	1.35	(1.18–1.54)	1.37	(1.16–1.60)
Striker	0.82	(0.75–0.88)	0.80	(0.71–0.90)	0.87	(0.76–1.00)	0.74	(0.64–0.86)	0.82	(0.69–0.98)	0.67	(0.52–0.86)
**Supervision**												
Level 1	3.01	(2.79–3.24)	2.77	(2.45–3.14)	2.81	(2.45–3.23)	1.19	(1.06–1.33)	1.28	(1.12–1.46)	3.15	(2.52–3.95)
Level 2	4.90	(4.45–5.40)	5.05	(4.32–5.89)	3.74	(3.12–4.48)	1.04	(0.89–1.23)	0.86	(0.70–1.06)	4.91	(3.70–6.50)
Level 3	3.31	(2.58–4.24)	3.19	(2.21–4.62)	3.23	(2.11–4.95)	0.77	(0.53–1.12)	0.97	(0.59–1.62)	2.00	(0.81–4.93)
Level 4	1.00	(reference)	1.00	(reference)	1.00	(reference)	1.00	(reference)	1.00	(reference)	1.00	(reference)
Level 5	2.91	(2.61–3.25)	3.15	(2.66–3.72)	2.76	(2.26–3.37)	0.83	(0.71–0.97)	0.83	(0.69–0.99)	2.62	(1.89–3.62)
**Offense Type**												
Drug	1.00	(reference)	1.00	(reference)	1.00	(reference)	1.00	(reference)	1.00	(reference)	1.00	(reference)
Violent	0.80	(0.74–0.86)	0.81	(0.73–0.91)	0.81	(0.71–0.92)	3.36	(2.87–3.93)	3.26	(2.71–3.92)	0.84	(0.68–1.03)
Property	1.20	(1.13–1.27)	1.17	(1.08–1.28)	1.20	(1.08–1.33)	5.78	(4.77–7.01)	5.42	(4.31–6.81)	1.15	(0.97–1.36)
Other	0.94	(0.87–1.03)	0.85	(0.74–0.98)	1.07	(0.92–1.24)	4.55	(2.83–7.31)	3.05	(1.58–5.90)	0.87	(0.68–1.11)
Sexual	0.80	(0.65–0.98)	0.73	(0.51–1.04)	0.73	(0.47–1.12)	2.87	(2.32–3.56)	2.88	(2.22–3.72)	1.00	(0.63–1.61)
**Parole Officer**												
Male Officer	0.95	(0.89–1.00)	0.95	(0.87–1.04)	0.93	(0.84–1.04)	0.93	(0.83–1.03)	1.01	(0.89–1.15)	0.92	(0.78–1.08)
Black Officer	1.06	(1.01–1.12)	1.04	(0.95–1.14)	1.10	(0.99–1.21)	1.13	(1.01–1.26)	1.05	(0.92–1.20)	0.95	(0.81–1.12)

Summary of stratified recurrent events Cox regression analysis for prison crowding and other covariates predicting parole violation rates.

* Results for prison crowding (and for other predictors of recidivism) were adjusted for: sex, race, mental health status, prior offense type, parole region, age first incarcerated, age when released, number of prior imprisonments, number of prior serious offenses, sex offender status, level of supervision, three-striker status, parole officer sex, and parole officer race. A Cox stratified procedure was used to adjust for covariates not meeting the proportional hazards assumption as well as those that did meet this assumption. These variables are sex offender status, parole region, age first incarcerated, age when released, number of prior imprisonments, and number of prior serious offenses.

### Adjusted results for specific violation types

Crowding level-dependent effects were found for each parole violation type (Table 2). The largest effects of high vs. low crowding were found for absconding violations (HR 3.56, 95% CI: 3.05–4.17) and Type I criminal violations (HR 2.44, 95% CI: 2.00–2.98). Other violation types—Type II criminal violations (HR 2.14, 95% CI: 1.73–2.64), Type III violations (HR 1.88, 95% CI: 1.45–2.43), and technical violations (HR 1.86, 95% CI: 1.37–2.53)—followed a similar pattern. Effect sizes for medium crowding compared to low crowding were proportionally smaller, and this data is also presented in Table 2.

Several of the confounders included in the model are shown in Table 2, including sex, race, history of mental illness, three striker status, level of supervision, offense type, and parole officer race and sex. Male sex and Black race were both associated with higher rates of parole violations, as was a history of mental illness. Parolees with three striker status had lower rates of parole violations than others (HR0.82, 95% CI: 0.75–0.88). Parole officer race, sex, and supervision level were also significantly associated with rates of parole violation, but as with the other confounders, all these effect sizes were comparatively small. Parolees who had been imprisoned for violent crimes had lower rates of combined parole violations than those who had been imprisoned for drug crimes (HR0.80, 95% CI: 0.74–0.86), whereas those who had been imprisoned for property crimes had higher rates of parole violations (HR 1.20, 95% CI 1.13–1.27). The latter association was particularly strong for Type II parole violations (HR 5.78, 95% CI 4.77–7.01), which include property crimes. Parolees who had been imprisoned for violent crimes were, however, more likely to have committed a Type II or Type III parole violation than those who had been imprisoned for drug crimes.

## Discussion

This study asked whether prison crowding is associated with higher rates of parole violations. Rates of parole violations were found to be directly associated with prison crowding levels, particularly for absconding violations and drug charges. The cause of parolee absconding is not always known. An indirect relationship with drug use is supported by some previous research [56, 57]. Other reasons for absconding could include seeking instrumental support from family and friends outside the parole region, or difficulty meeting parole conditions such as finding and maintaining local employment. After absconding, the second largest effect was seen in Type I criminal violations, which are considered to be the least serious and are almost entirely drug-related.

Substance use disorder (SUD), which is overrepresented in prison populations, may be inadequately treated in crowded prisons, increasing the risk of drug-related recidivism for this subgroup of parolees. Almost a third of recidivists return due to drug offenses [58]. In this respect, SUD-related violations represent a major vulnerability within the parole system. For instance, in Ohio and Texas, former prisoners with SUD are three times more likely to return to prison one year after release [59]. Others have found that parolees with SUDs that are comorbid with other psychiatric conditions have a significantly higher likelihood of returning to prison within one year after release [60–61]. Nationally, 42.8% of individuals with SUDs also have had a comorbid psychiatric disorder [62].

Smaller magnitude-dependent effects were also seen for Types II and III violations. Type II violations include moderately serious drug offenses, such as sales and trafficking, but they also include property offenses, consensual sex offenses, and other non-violent offenses. Type III violations are serious violent and sexual offenses. The association with Type III violations was large enough to warrant further research on the impact crowding might have on public safety (e.g., rape, homicide, burglary, child abuse). This finding is consistent with research that reports that crowding induced violence against inmates is brutalizing in nature and may have an unintended violent criminogenic effect [63]. The smallest effect size was seen with technical violations. Such events are not normally associated with criminal sanctions, and they include behaviors such as missing appointments, traveling outside a parole boundary, accessing legal weapons, and other non-criminal parole conditions.

The relationship between prison crowding and recidivism may be mediated by stress hormones such as cortisol, which is associated with impaired executive function in children exposed to crowding-related stress [13]. An impairment in executive function has also been shown in parents exposed to residential crowding, and in patients with post-traumatic stress disorder [14, 64]. Alternatively, executive function may also be impaired from drug use in those with active SUD, who may have limited access to drug treatment in more crowded prisons. However, the literature on adverse effects of prison crowding on inmate health and behavior is sparse, and the literature on crowding, executive function and aggression is most extensive for studies on childhood behavior and development [65]. Only studies on children show adverse effects from crowding that are measureable after research subjects have been exposed to the crowded environment, as opposed to within crowded conditions [13, 15]. Strong claims about cause and effect are impossible without further research, particularly on the mediating roles of cognition, social environment and coping skills.

The relationship between prison crowding and recidivism may also be mediated by infectious, chronic and non-substance related psychiatric conditions that contribute to incarceration. Crowding has been repeatedly linked to high blood pressure levels in prison populations, the prison prevalence of infectious diseases is substantially higher than the national average, and upon release from prison, parolees have decreased access to care [65, 6, 66, 67]. The strain, monetary and otherwise, of an added disease burden related to exposure to prison crowding could lead to an increased parole violation rate. Due to limitations in available data, information about individual parolees’ physical health conditions was not available. However, information pertaining to mental health conditions was available. The results indicate that parolees with this designation had higher rates of parole violations (of all types) than those without a mental health designation (HR 1.46 95%, CI: 1.38–1.55). As previously discussed, there may be an interaction in parolees with co-occurring disorders.

The potential for an effect of increased recidivism risk for parolees with chronic conditions is still possible. However, past research has shown only a very weak correlation between parolees with chronic physical conditions and a greater risk for recidivism [68]. These parolees tend to be older, and age is a known confounder for recidivism. Further research is needed among this older population.

### Limitations

This study has several limitations. First, weighted averages for crowding measures were aggregated at the level of parole region for each observation point. The potential of an ecological fallacy bias arises for this reason, although it has been mitigated by hybridizing individual-level data into multi-level models [69]. For example, if residency in Los Angeles County or Southern California carries additional risk factors for higher rates of parole violations, aggregating crowding data by region would introduce important confounders to the model. Hybrid multi-level modeling mitigates this problem by nesting individual-level data with ecological variables, so that, in this case, risk factors for parole violation that vary by region (e.g., supervision level, race) were captured at the individual level. Any number of missing variables may act as ecological confounders, such as regional variation in levels of access to SUD treatment outside the prison system, or regional variation in the intensity of the drug trade.

Second, more than 15% of the values were missing for the following variables: age at release (15.6%), parole region (15.6%), days since release (20.5%), and supervision level (35%). The reasons for missing data are unknown and may, therefore, be non-random. A random recursive partitioning method (described in the methods section) for missing data imputation was used to help lessen potential bias. Only data on violation events were imputed, and it should be noted that the vast majority of missing data on parole region, age at release and days since release was for non-violation observations that were not censored. Because there were much higher rates of missing data for censored events on supervision level, parole officer race and parole officer sex for observation week 105 only (the final observation week in the data set), the imputation was done using data through week 104 only.

Third, we do not know how the data set was censored before it was made available to us. Overall, the data was left-truncated and right-censored, but we do not have a description of how mortality was handled in the censoring. This is an important limitation to our paper, as we are unable to report tests on informative censoring that would shed light on a potential source of bias in our analysis.

It is possible that crowding does not significantly increase the risk of recidivism among California parolees. Risk factors outside of the criminal justice system (e.g., poverty, child abuse history, employment status, familial integrity) could potentially overshadow the effect of crowding; if they vary by region within the state of California, they represent important unmeasured confounders with crowding in this ecological analysis. The influence of social determinants of health (e.g., poverty, county discipline, public assistance) on rates of recidivism could not be included in our analysis because the percentage of missing values was too high (>40%) for the data to be imputed. Information on race was limited to a dichotomous variable (Black or non-Black). Important confounders with the demographic variables race, age and disability (mental illness), such as socioeconomic status and education level, could not be included in the model because of the missing data, making the interpretation of the results for demographic variables somewhat ambiguous. Low socioeconomic status, limited access to public assistance, low education levels and an adverse social environment are all risk factors for both SUD and incarceration that may be overrepresented in regions with higher rates of prison crowding.

Surveillance bias or differences across regions in quantitative enforcement activity targets may also have biased the results. Quotas and other similar police management tools have been shown to strongly predict arrest rates even where measures have been taken to preserve officer discretion and prevent arbitrary arrests [70]. Many parole agents attest that parole violations are highly subjective, that is, that "a parole agent can always find a violation for a parolee if he or she wants to" [20]. Surveillance bias could also explain why all types of parole violations, including technical violations, were directly associated with prison crowding.

Unfortunately, history of SUD was only measured at the aggregate level in this data set, and access to drug treatment in prison was not measured at all. If prison crowding prevents some inmates with SUD from receiving adequate drug treatment prior to release, this could be an important predictor of drug-related parole violations. Our aggregate level data on this prison population shows that SUD is highly prevalent in this sampling frame, so this represents another important uncontrolled confounder in our analysis—we do not have data on whether or not SUD prevalence varies by region among California parolees.

Most of the parolees in this study had just served less than five months in prison, making the control variables for prior history of imprisonment important covariates in our model. The adjusted analysis controlled for age at first incarceration, number of prior offenses leading to imprisonment ("number of returns to prison" in Table 1), number of prior serious offenses, prior offense type and three-striker status. There may, however, be important related confounders not included in the model, such as total duration of time in prison inclusive of all prior offenses.

Finally, the Cox proportional hazards model assumes that observations among individuals are independent. This study’s data involved repeated events within subjects. Although the beta coefficients in the study models were not adjusted, the variances were adjusted for using an empirical estimator. These adjustments allowed for comparisons between the hypotheses and their confidence intervals [51]. However, robust error variance estimation does not capture heterogeneity among parolees in rates of recidivism; instead, this method considers within-subject event likelihood correlation to be a nuisance variable. Offense type was an important confounder in our model, strongly predictive of parole violation type in adjusted analysis. This does suggest within-subject correlation in propensity to violate parole.

### Study Implications

Previous corrections studies have posited that adverse prison conditions act to deter freed prisoners from returning to prison. The present findings do not support this deterrent model of corrections, instead agreeing with other findings [22, 26]. Additional criminal justice approaches to drug use, such as arrests, have similarly been found to be counterproductive [71]. Average outpatient drug treatment in the United States costs 14.4 times less, and long-term residential drug treatment costs 3.8 times less, than incarceration [72]. In 2010, CDCR reported that the annual cost of incarcerating a single prisoner was $45000 per year. Additionally, states with higher than national average drug treatment rates send approximately 100 fewer people to prison per 100000 than states with below national average drug treatment rates [73]. Mental health courts are also considered an important means of redressing prison crowding; a shortage of beds in inpatient psychiatric wards is directly related to the high prevalence of mental illness in prison populations [74].

The relationship between mass incarceration and crowding-related stress as well as prison violence and lack of access to SUD care may explain the finding that parolees who experienced higher levels of prison crowding had higher rates of parole violations, and that this association was particularly strong for drug-related parole violations. Potential interventions include measures to redress both prison crowding and negligent SUD and mental health care, such as the use of mandatory drug treatment referrals as an alternative to incarceration, and increased use of mental health courts. Further research on the association between prison crowding and recidivism is warranted.

### Future Areas of Research

#### Adverse prison experiences

Further research on negligent SUD care and prison violence would be needed to better understand the relationship between prison crowding and both PTSD and SUD. In addition, data on drug-related parole violations are no substitute for direct measures of SUD relapse among parolees. Additional research on the relationship between access to drug treatment in prison and rates of SUD relapse is warranted.

#### County and city jails

A recent criminal justice reform (known as the Public Safety Realignment Act), charges counties and municipalities with additional responsibilities, including; supervision of most state parolees and incarceration of most newly convicted persons, as well as parole violators [75]. Little is known about the potential effect that such a large burden on county facilities could have. Many counties, similar to the state, have opted to construct more detention facilities [76]. As a whole California jails already are over capacity [77]. More information about the effects of overcrowding in county jails is currently needed.

## Supporting Information

S1 File Composited Data Set. (CSV)
